# Ileum terminal antibiotic infusion affects jejunal and colonic specific microbial population and immune status in growing pigs

**DOI:** 10.1186/s40104-018-0265-x

**Published:** 2018-07-02

**Authors:** Chuanjian Zhang, Yu Peng, Chunlong Mu, Weiyun Zhu

**Affiliations:** Jiangsu Key Laboratory of Gastrointestinal Nutrition and Animal Health, Laboratory of Gastrointestinal Microbiology, College of Animal Science and Technology, Nanjing Agricultural University, National Center for International Research on Animal Gut Nutrition, Nanjing, 210095 China

**Keywords:** Antibiotic infusion, Growing pigs, Gut bacteria, Immune status, Short-chain fatty acids

## Abstract

**Background:**

Compared with oral antibiotics (primarily disrupt foregut microbiota), the present study used antibiotics with ileum terminal infusion to disrupt the hindgut microbiota, and investigated the changes in specific bacterial composition and immune indexes in the jejunum and colon, and serum of growing pigs. Twelve barrows (45 d of age, 12.08 ± 0.28 kg) fitted with a T-cannula at the terminal ileum, were randomly assigned to two groups and infused either saline without antibiotics (Control) or with antibiotics (Antibiotic) at the terminal ileum. After 25 d experiment, all pigs were euthanized for analyzing bacterial composition and immune status.

**Results:**

Ileum terminal antibiotic infusion (ITAI) altered dominant bacteria counts, with a decrease in *Bifidobacterium*, *Clostridium* cluster IV and *Clostridium* cluster IV in the colon (*P* < 0.05), and an increase in *Escherichia coli* in the jejunum (*P* < 0.05). ITAI decreased (*P* < 0.05) short-chain fatty acids concentrations in the colon. ITAI decreased interleukin-8 (IL-8), IL-10 and secretory immunoglobulin A (sIgA) concentrations, and down-regulated *IL-10*, Mucin-1 (*MUC1*), Mucin-2 (*MUC2*) and zonula occludens-1 (*ZO-1*) mRNA expression in the colonic mucosa (*P* < 0.05). In the jejunal mucosa, ITAI decreased interferon-γ (IFN-γ), tumor necrosis factor-α (TNF-α), sIgA and IgG levels together with down-regulation of *IFN-γ*, *TNF-α*, *MUC2* and *ZO-1* mRNA expression (*P* < 0.05). Furthermore, ITAI decreased IL-10, INF-γ, TNF-α, IgA and IgG concentrations in serum (*P* < 0.05). Correlation analysis revealed that the change in intestinal microbiota was correlated with alterations of Ig and cytokines.

**Conclusions:**

ITAI affected jejunal and colonic specific bacteria counts, and altered some immune markers levels in the jejunal and colonic mucosa and serum. These findings implicate the potential contribution of hindgut bacteria to immune response in the intestinal mucosa and serum of growing pigs.

**Electronic supplementary material:**

The online version of this article (10.1186/s40104-018-0265-x) contains supplementary material, which is available to authorized users.

## Background

The large intestine of pigs constitutes an environment for the development and activity of the largest microbiota population in the body, which affects the resistance to disease, function of the gastrointestinal tract and health status. Resident bacteria play a pivotal role in development and shaping of the immune system. Loss of diversity and shift in community composition in large intestine may suppress the host’s innate immune defense, contributing to disease susceptibility [1]. Meanwhile, microbial metabolites could also affect mucosal immune response [2]. Short-chain fatty acid (SCFA), which are produced mainly from the microbial fermentation of carbohydrates, are considered to be beneficial to the host intestine as they provide energy for epithelial cells, inhibit potential pathogen growth, stimulate epithelial proliferation, facilitate tight junction formation and inhibit inflammation and genotoxicity [3–5]. Furthermore, the immune system affords mammalian hosts some control over the composition of their resident microbial communities [6]. This leads to the idea of the immune system as a form of ecosystem management that exerts critical control over microbiota composition, diversity, and location [7]. Therefore, a symbiotic relationship between the host and the microbiota maintains intestinal homeostasis and prevents intestinal disease.

It is well known that compared to microbiota in the small intestine, microbiota in the hindgut have higher diversity and cell densities [8]. Besides, microbial fermentation in pigs occurs to the largest extent in the hindgut [9]. This enriched microbiota and microbial fermentation products in the hindgut are important to intestinal immune system [10]. Furthermore, previous studies have demonstrated cross-talks between colonic immunity with serum immunity or small intestinal immunity [11, 12]. Therefore, it is important to understand the effect of hindgut microbiota perturbation on gut (small intestine and large intestine) and serum immunity.

The most profound disrupters of the gut microbiome are antibiotics. Our recent study demonstrated that in feed antibiotics (olaquindox, oxytetracycline calcium and kitasamycin) exerted profound impact on bacteria in the small intestine and less impact on bacteria in colon of piglets [13]. Furthermore, oral chlortetracycline altered ileal microbiota with a decrease in the abundance of *Lactobacillus johnsonii*, Clostridiales and *Turicibacter* and an increase in the abundance of *Lactobacillus amylovorus* [14], but had limited effect on fecal microbiota of pig [15]. These results suggest that in feed antibiotics primarily disrupt the foregut microbiota. Oral antibiotics influenced intestinal and systemic immune parameters [16]. Oral antibiotics reduced mucosal interleukin-17A (IL-17A) and interferon-γ (IFN-γ) production by CD4^+^ T lymphocytes in the small intestine [17], and *MUC2* mRNA expression in the colon of mice [1, 18]. Oral amoxicillin and clavulanate potassium reduced IgG concentration in the serum of healthy adult humans [19]. Compared with oral antibiotics (primarily disrupt foregut microbiota), the present study used antibiotics with ileum terminal infusion to perturb the hindgut bacteria, to investigate the changes in specific immune responses of gut and serum in pigs. We hypothesized that ileum terminal antibiotic infusion (ITAI) decreased SCFA-producing bacteria counts and SCFA concentration in the colon, which is different from oral antibiotics [13, 20]. We also hypothesized that ITAI altered some markers of immunity in the intestinal mucosa and serum with a decrease in the levels of specific inflammatory cytokines and immunoglobulins, which is similar to oral antibiotics [17, 19]. The findings may provide further insights into our understanding of the impact of hindgut microbiota perturbation on gut (small intestine and large intestine) and serum immunity.

## Methods

### Animals, housing, diets, surgery and infusions

Twelve 35-day-old growing barrows (Duroc × Landrace × Large White, weaned at 21 d with body weight of 10.00 ± 0.5 kg) were obtained from a commercial farm in Jiangsu Province of China. All pigs were fitted with a simple T-cannula in the distal ileum [21] and jugular catheters [22]. After surgery, pigs were housed individually in smooth-sided metabolic crates in a temperature-controlled room at 28 °C with free access to water and allowed a 10-d recovery period. Pigs were fed increasing amounts of a 19% crude protein (CP) commercial feed during the 5-d of recovery period. Thereafter, all pigs received the corn-soybean diet formulated based on NRC requirements without any antibiotic cocktail (Table 1) [23]. At d 45 of age, 12 pigs (12.08 ± 0.28 kg) were randomly assigned to 1 of 2 infusion treatment based on equal body weights: 1) control (*n* = 6) receiving infusion of 10 mL saline solution (0.9% NaCl) via T-cannula; 2) antibiotic (*n* = 6) receiving infusion of 10 mL saline solution with antibiotic (ampicillin, 150 (mg/kg)/d, gentamicin, 4 (mg/kg)/d, and metronidazole, 30 (mg/kg)/d) via T-cannula. Solutions were infused every morning before feeding, and the concentration of the infusion solution was adjusted for every 3-d according to the body weight. Ampicillin is one of β-lactam antibiotics with activity against Gram-positive and Gram-negative bacteria. Gentamycin exhibits activity mainly against most Gram-negative bacteria and anaerobes. Metronidazole can act against anaerobes. This antibiotic cocktail acts against most bacteria in the gastrointestinal tract of pigs. After 25 d experiment, all pigs were euthanized. Body weight and feed consumption of individual pigs were recorded from d 45 of age to d 70 of age in order to determine average daily gain (ADG), average daily feed intake (ADFI), and gain to feed ratio (G:F). Blood samples (10 mL) were collected from the jugular vein of pigs and serum was obtained by centrifuging at 3,000×*g* for 10 min at 4 °C. Thereafter, the serum samples were stored at − 20 °C for determination of immunoglobulin (Ig) and cytokines. Contents of jejunum and colon were collected and stored at − 80 °C for later bacterial DNA extraction. In addition, a small piece of gut tissue from middle section of jejunum and colon was excised and rinsed in PBS. The mucosa scrapings were collected by scraping off the mucosa using a sterile glass microscope slide and immediately stored at − 80 °C for RNA, cytokines and sIgA analyses.

**Table 1:** Composition and analyzed nutrient contents of experimental diets (as-fed basis)

Item	Content
Ingredient, %	
Corn	69.17
Soybean meal	19.40
Soybean protein concentrate	4.00
Soybean oil	0.50
Fish meal	3.00
*L*-Lysine HCl, 98.50%	0.35
*DL*-Methionine	0.18
*L*-Threonine	0.13
*L*-Tryptophan	0.02
Stone dust	0.80
Dicalcium phosphate	0.75
Sodium chloride	0.30
Choline chloride	0.10
Chromic oxide	0.30
Vitamins and minerals Premix^a^	1.00
Total	100.00
Calculated composition, %	
NE, kcal/kg	2554.75
CP,%	19.19
EE,%	4.52
CF,%	2.15
Ca,%	0.65
P, %	0.44
SID^b^ Lysine	1.16
SID Methionine	0.46
SID Tryptophan	0.21
SID Threonine	0.71
Analyzed composition, %	
CP	19.10
Fat	4.28

^a^Supplied the following per kg of diet: 8,000 IU, vitamin A; 2,400 IU, vitamin D_3_; 20 mg, vitamin E; 5 mg, vitamin B_6_; 0.03 mg, vitamin B_12_; 15 mg, pantothenic acid; 0.3 mg, biotin; 3 mg, folic acid; 40 mg, ascorbic acid; 120 mg, Fe; 25 mg, Cu; 20 mg, Mn; 150 mg, Zn; 0.5 mg, I; 0.30 mg, Se.

^b^*SID* standardized ileal digestible

### Microbial DNA isolation and real-time qPCR analysis of the bacterial 16S ribosomal RNA gene

Total genomic DNA was extracted from 300 mg fresh jejunal and colonic luminal contents by using a bead-beating method and phenol-chloroform extraction [24]. The DNA was then precipitated with ethanol and the pellets were dissolved in 80 μL of Tris EDTA (TE).

Numbers of total bacteria, *Bacteroides-Prevotella*, *Bifidobacterium*, *Clostridium* cluster IV, *Clostridium* cluster XIVa, *Escherichia coli* and *Lactobacillus* were quantified by real-time polymerase chain reaction (PCR) using specific primers (Table 2) and SYBR Green Premix (Takara Biotechnology, Dalian, China) in the StepOnePlus™ Real-Time PCR System (Life Technologies, California, USA). Quantification of 16S rRNA gene copies in each sample was performed in triplicate, and the mean value was calculated. Each bacterial group copy number in the jejunum or colon was quantified according to standard curve of bacterial groups which were generated from serial dilutions of the plasmid. For each reference strain, target gene (the 16S rRNA gene) was cloned into a pMD-19 T Vector System (TaKaRa Biotechnology, Dalian, China). An *Escherichia coli* strain was transformed with the recombinant plasmid, and plasmid DNA was extracted from *Escherichia coli* by the miniprep method [25].

**Table 2:** List of primers used in this study

Bacteria	Forward primer, 5′→3’	Reverse primer, 5′→3’	Reference	Annealing temp, °C
Total bacteria	GTGSTGCAYGGYYGTCGTCA	ACGTCRTCCMCNCCTTCCTC	[63]	60
*Bacteroides-Prevotella*	GAGAGGAAGGTCCCCCAC	CGCTACTTGGCTGGTTCAG	[64]	60
*Bifidobacterium*	TCGCGTCYGGTGTGAAAG	GGTGTTCTTCCCGATATCTACA	[65]	60
*Clostridium* cluster IV	GCACAAGCAGTGGAGT	CTTCCTCCGTTTTGTCAA	[66]	60
*Clostridium* cluster XIVa	CGGTACCTGACTAAGAAGC	AGTTTYATTCTTGCGAACG	[67]	60
*Escherichia coli*	CATGCCGCGTGTATGAAGAA	CGGGTAACGTCAATGAGCAAA	[68]	60
*Lactobacillus*	AGCAGTAGGGAATCTTCCA	ATTCCACCGCTACACATG	[69]	60

### Short-chain fatty acid in the jejunum and colon

Jejunal and colonic digesta were prepared for total short-chain fatty acid (SCFA) analysis by mixing 0.4 g of digesta with 0.2 mL of 25% (*w*/*v*) metaphosphoric acid and 1.6 mL of water. The mixture was vortexed and then centrifuged at 13,000×*g* for 10 min at 4 °C. The supernatant was frozen at − 20 °C and used for SCFA determination [26].

### Reverse transcription quantitative PCR analysis of jejunal and colonic epithelial gene expression

Total RNA was extracted from jejunal and colonic mucosa with using TRIzol reagent (Takara Bio, Otsu, Japan) [27]. The RNA concentration was then quantified using a NanoDrop spectrophotometer (ND-1000UV-Vis; Thermo Fisher Scientific, Waltham, MA), and the absorption ratio (260/280 nm) ranging from 1.8 to 2.0 indicated high RNA purity. Complementary DNA (cDNA) was reverse transcribed from 1 μg of eluted RNA by using a PrimeScript® RT Reagent Kit with gDNA Eraser (Takara Bio) following the manufacturer’s instructions.

Expression levels of toll-like receptor (*TLR2*, *TLR4* and *TLR5*), intestinal cytokines [*IL-8*, *IL-10, IFN-γ* and tumor necrosis factor-α (*TNF-α*)], chemical barrier function (Mucin-1 and Mucin-2) and mechanical barrier function [Occludin and zonula occludens-1 (*ZO-1*)] were analyzed by real-time quantitative PCR with SYBR Green PCR reagents (TaKaRa), and analyses were performed using the ABI 7300 Real-time PCR system (Applied Biosystems, Foster, CA, USA). The specific primers used in this study were listed below (Table 3). Amplification conditions were as follows: 95 °C for 30 s, followed by 40 cycles composed of 5 s at 95 °C and 30 s at 60 °C. All measurements were made in triplicate, and the mean threshold cycle was calculated. The results were normalized to the expression of *β-actin* gene and relative expression levels were calculated by using the 2^-ΔΔCt^ method.

**Table 3:** Primer pairs for host genes

Gene^a^	Forward primer, 5′→3’	Reverse primer, 5′→3’	Reference	Annealing temp, °C
*β-actin*	AGAGCGCAAGTACTCCGTGT	ACATCTGCTGGAAGGTGGAC	[70]	60
*TLR2*	TCACTTGTCTAACTTATCATCCTCTTG	TCAGCGAAGGTGTCATTATTGC	[71]	60
*TLR4*	TCAGTTCTCACCTTCCTCCTG	GTTCATTCCTCACCCAGTCTTC	[72]	60
*TLR5*	CAGCGACCAAAACAGATTGA	TGCTCACCAGACAGACAACC	[71]	60
*IL-8*	ACTGGCTGT TGCCTTCTT	CAGTT CTCTTCAAAAATATCTG	[73]	60
*IL-10*	GTCCGACTCAACGAAGAAGG	GCCAGGAAGATCAGGCAATA	[70]	60
*IFN-γ*	TCCAGCGCAAAGCCATCAGTG	ATGCTCTCTGGCCTTGGAACATAGT	[74]	60
*TNF-α*	CCACGCTCTTCTGCCTACTGC	GCTGTCCCTCGGCTTTGAC	[73]	60
*MUC1*	GGTACCCGGCTGGGGCATTG	GGTAGGCATCCCGGGTCGGA	[70]	60
*MUC2*	CTGCTCCGGGTCCTGTGGGA	CCCGCTGGCTGGTGCGATAC	[70]	60
*OCLN*	ATGCTTTCTCAGCCAGCG TA	AAG GTTCCATAGCCTCGGTC	[73]	60
*ZO-1*	GAGGATGGTCACACCGTGGT	GGAGGATGCTGTTGTCTCGG	[73]	60

^a^*TLR2* toll-like receptor2, *TLR4* toll-like receptor 4, *TLR5* toll-like receptor 5, *IL-8* interleukin-8, *IL-10* interleukin-10, *IFN-γ* interferon-γ, *TNF-α* tumor necrosis factor-α, *ZO-1* zonula occludens-1, *MUC1 * mucin-1; *MUC2*  mucin-2; *OCLN * occludin

### Immunoglobulins and cytokines analyses

Accordance with the manufacturer’s instructions, porcine-specific ELISA kits were used to quantify circulating immunoglobulin (IgA and IgG) (Bethyl Laboratories, Montgomery, TX). Immunoglobulin (sIgA and IgG) in the mucosa and cytokines (IL-8, IL-10, TNF-α and IFN-γ) in the serum and mucosa were measured using commercially available ELISA kits (Beijing 4A BiotechCo., Ltd., Beijing, China) in accordance with the manufacturer’s instructions and run in duplicates. Before the assays, the mucosa samples were vortexed in PBS (1:10, *w*/*w*) for 60 s and centrifuged at 5,000×*g* for at 4 °C, and the supernatant was obtained and used for the determination of cytokines and Ig levels. The concentrations of each cytokine and Ig in the intestinal mucosa were standardized to the protein in each sample. Total protein content in the mucosa was determined with Coomassie blue method following the manufacturer’s instructions (Nanjing Jiancheng Bioengineering Institute, Nanjing, China).

### Statistical analysis

Data were analyzed by SPSS 16.0 (SPSS Inc., Chicago, IL, USA) as a randomized block design, considering the antibiotic as main effect and the replicate as a block. The individual pig was used as the experimental unit (*n* = 6) of all analysis. Results were statistically analyzed by Student’s *t*-test corrected with the false-discovery rate (FDR). All data were expressed as mean ± SEM, and *P* ≤ 0.05 was considered statistically significant. The *R* package of “Hmisc” was used for calculating the spearman’s correlation coefficient.

## Results

### Growth performance

During the whole experiment period, no clinical signs of diarrhoea or health impairment were observed with all pigs. Average daily gain did not significantly differ (*P* > 0.05) between the antibiotic group and control group (483.26 ± 57.82 vs. 539.16 ± 69.50 g/d). There was no significant difference (*P* > 0.05) in the average daily feed intake between the antibiotic group and control group (952.40 ± 49.85 vs. 1046.89 ± 61.58 g/d). Furthermore, no significant differences (*P* > 0.05) in feed:gain were observed between the antibiotic group and control group (2.07 ± 0.18 vs. 2.08 ± 0.29) (data not shown).

### Dominant microbial groups in jejunal and colonic contents

**Fig. 1:**
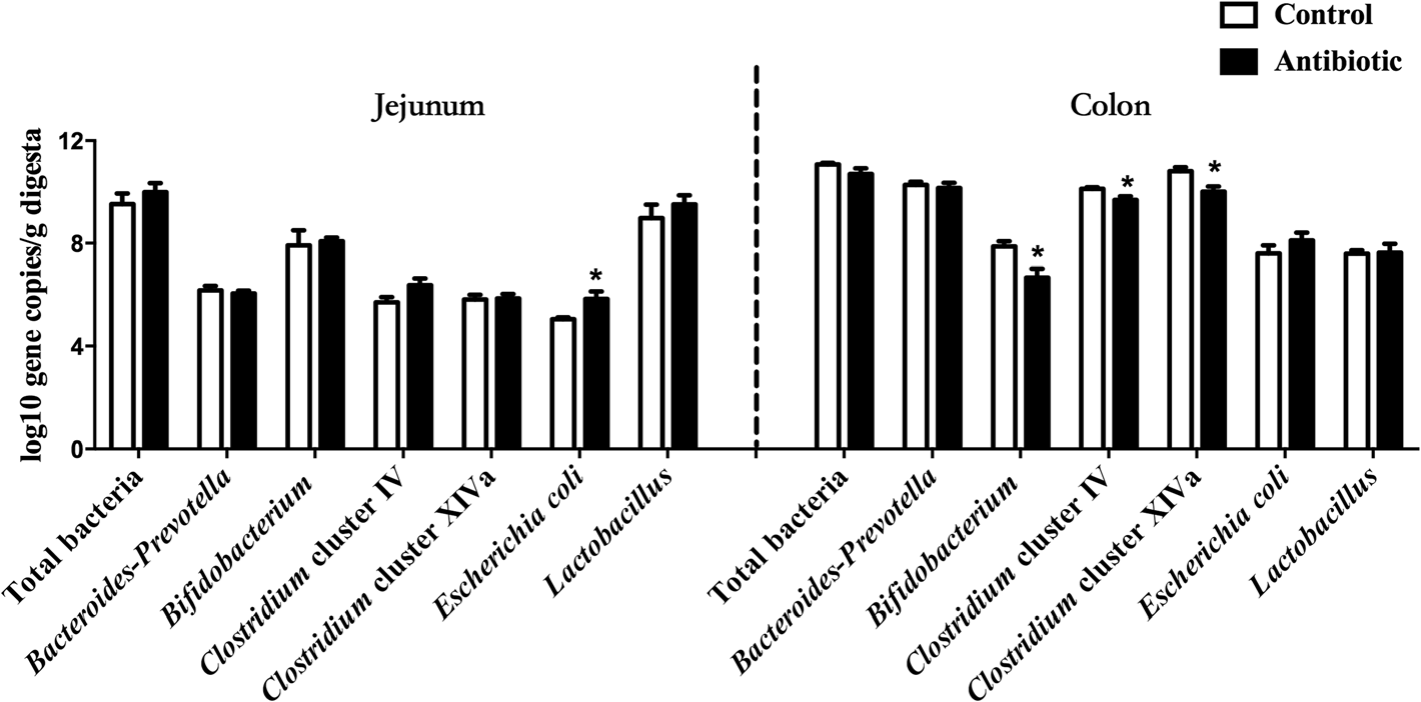
Effects of ileum terminal antibiotic infusion (ITAI) on copy numbers (Lg [copies/g]) of major bacterial taxonomic groups in the jejunum and colon of pigs. Values are means ± SEMs (*n* = 6). Asterisks indicate statistically significant difference from control: *P < 0.05

The results of the effect of ITAI on intestinal dominant microbiota determined by real-time PCR are shown in Fig. 1. Total bacterial counts detected in the jejunum and colon were similar between the antibiotic and control groups. In the jejunum, ITAI significantly increased *Escherichia coli* counts (*P* < 0.05). However, the counts of *Bacteroides-Prevotella, Bifidobacterium, Clostridium* cluster IV, *Clostridium* cluster XIVa and *Lactobacillus* did not significantly differ between the antibiotic and control groups. In the colon, ITAI significantly decreased (*P* < 0.05) *Bifidobacterium, Clostridium* cluster IV and *Clostridium* cluster XIVa counts, but had little effect on *Bacteroides-Prevotella*, *Lactobacillus* and *Escherichia coli* counts. These results indicate that ITAI significantly changed particular bacterial species in growing pigs, with a decrease in the counts of generally beneficial bacteria in the colon and an increase in *Escherichia coli* counts in the jejunum.

### Short chain fatty acid

The concentrations of SCFAs were measured as indicators of microbial fermentation. In the jejunum, SCFAs were below the limit of detection in both groups (data not shown). In the colon, ITAI significantly decreased (*P* < 0.05) the concentrations of propionate, isobutyrate, butyrate, total SCFA, and tended to decrease (*P* = 0.054 and *P* = 0.075, respectively) the concentrations of acetate and isovalerate (Table 4). However, valerate concentration did not differ significantly between the antibiotic and control groups. These results indicate that ITAI significantly decreased the production of the beneficial compound SCFA in the colon.

**Table 4:** Effects of ileum terminal antibiotic infusion (ITAI) on short chain fatty acid (SCFA) concentrations in colon of pigs^a^

Item^b^, μmol/g digesta	Control	Antibiotic	*P-*value
Acetate	65.68 ± 5.83	48.76 ± 5.08	0.054
Propionate	27.71 ± 2.62	19.32 ± 2.09	0.031
Isobutyrate	0.64 ± 0.10	0.33 ± 0.08	0.034
Butyrate	9.33 ± 0.88	4.06 ± 1.06	0.003
Isovalerate	1.04 ± 0.18	0.64 ± 0.07	0.075
Valerate	1.14 ± 0.10	0.62 ± 0.29	0.115
Total SCFA	105.55 ± 8.26	73.73 ± 7.06	0.015

^a^Values are mean ± SEMs; *n* = 6 /group

^b^Total SCFA = total short-chain fatty acid

### Gene expression analysis in the mucosa of jejunum and colon

**Fig. 2:**
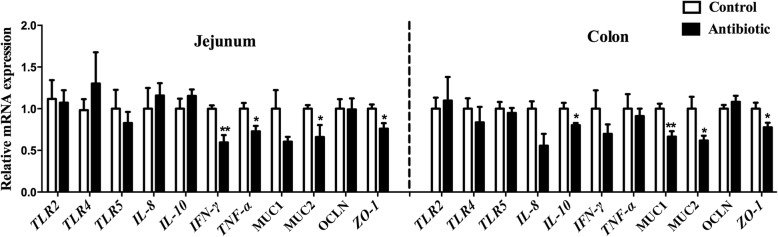
Effects of ileum terminal antibiotic infusion (ITAI) on the mRNA expression of genes related to TLRs, cytokines and barrier function in the jejunal and colonic mucosa of growing pigs. Values are means ± SEMs (*n* = 6). Asterisks indicate statistically significant difference from control: *P < 0.05, **P < 0.01. *TLR2* = toll-like receptor 2; *TLR4* = toll-like receptor 4; *TLR5* = toll-like receptor 5; *IL-8* = interleukin-8; *IL-10* = interleukin-10; *IFN-γ* = interferon-γ; *TNF-α* = tumor necrosis factor-α; *ZO-1* = zonula occludens-1; *MUC1 *= mucin-1; *MUC2* = mucin-2; *OCLN* = occludin

Effects of ITAI on genes expression in jejunal and colonic mucosa are shown in Fig. 2. There was no effect of ITAI on mRNA expression of innate immune receptors (*TLR2*, *TLR4* and *TLR5*) in the mucosa of jejunum and colon. For pro-inflammatory cytokines (*IL-8*, *IFN-γ* and *TNF-α*) and anti-inflammatory cytokines (*IL-10*), in the jejunal mucosa, ITAI significantly decreased (*P* < 0.05) *IFN-γ* and *TNF-α* mRNA expression, while mRNA expression of *IL-8* and *IL-10* remained similar in both experimental groups. In the colonic mucosa, *IL-10* mRNA expression was significantly decreased in response to the antibiotics (*P* < 0.05). However, no significant changes of *IL-8*, *IFN-γ* and *TNF-α* gene expression measured by qRT-PCR were recorded between the antibiotic and control groups. For chemical barrier function (Mucin-1 and Mucin-2) and mechanical barrier function (Occludin and ZO-1), in the jejunal mucosa, no differences in the mRNA expression of *MUC1* and *OCLN* were observed between the antibiotic and control groups, but ITAI significantly decreased (*P* < 0.05) *MUC2* and *ZO-1* mRNA expression. In the colonic mucosa, the mRNA expression of *MUC1*, *MUC2* and *ZO-1* originating from the antibiotic group were significantly decreased (*P* < 0.05) compared to control group. These results indicate that ITAI down-regulated specific genes involved in intestinal immunity, with different effect between jejunum and colon.

### Immunoglobulin and cytokines concentrations in the mucosa of jejunum and colon

**Fig. 3:**
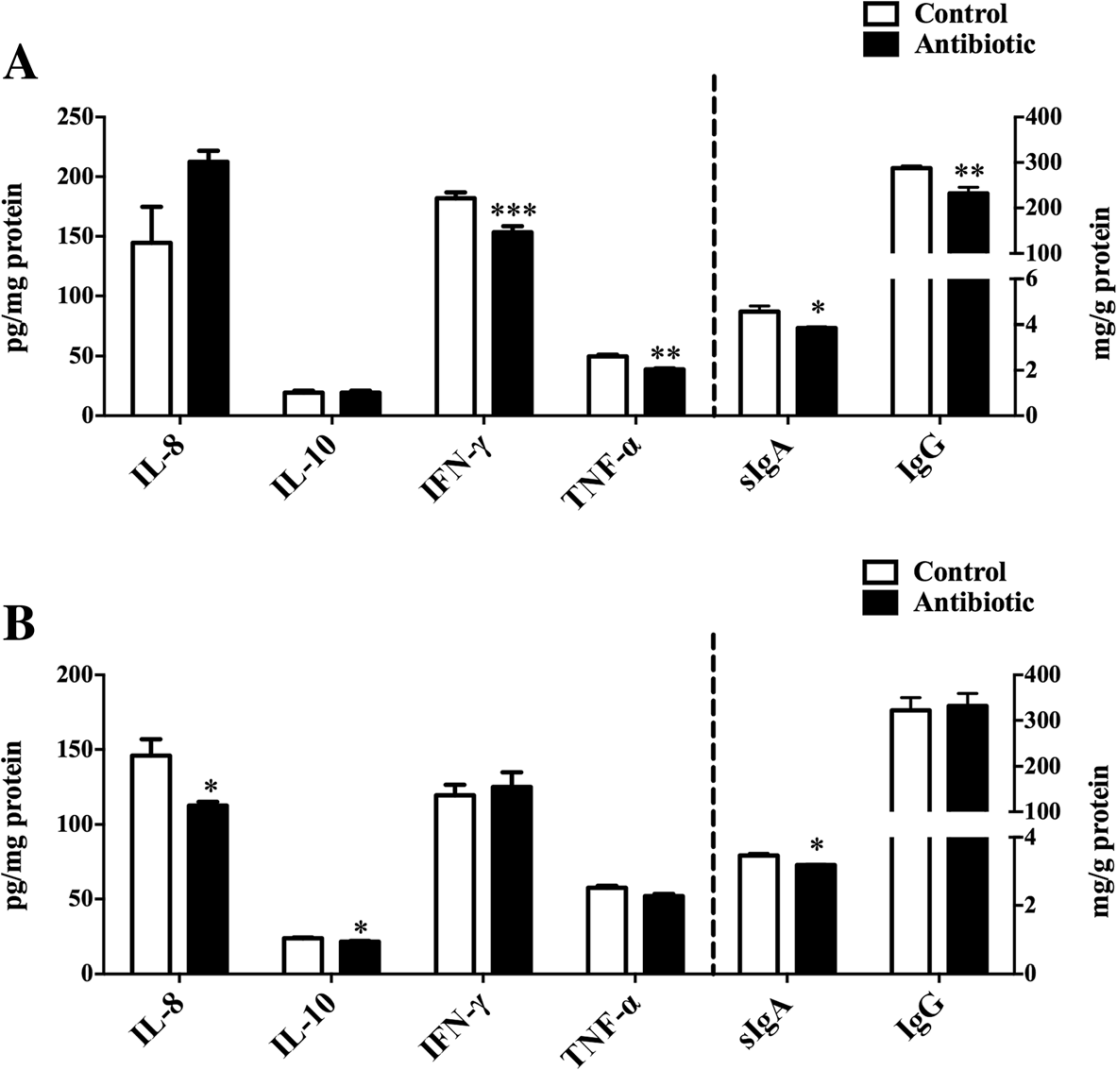
Effect of ileum terminal antibiotic infusion (ITAI) on the concentrations of intestinal cytokines and immunoglobulins in the jejunum (**a**) and colon (**b**) of pigs. Values are means ± SEMs (*n* = 6). Asterisks indicate statistically significant difference from control: *P < 0.05, **P < 0.01, ***P < 0.001. IL-8 = interleukin-8; IL-10 = interleukin-10; IFN-γ = interferon-γ; TNF-α = tumor necrosis factor-α; sIgA = secretory immunoglobulin A; IgG = immunoglobulin G

The results of the effects of ITAI on jejunal and colonic Ig and cytokines concentrations by ELISA are shown in Fig. 3. For cytokines (IL-8, IL-10, IFN-γ and TNF-α), in the jejunum, IFN-γ and TNF-α concentrations originating from the antibiotic group were significantly decreased compared to control group (*P* < 0.05, Fig. 3a). However, no significant differences in IL-8 and IL-10 concentrations were observed between the antibiotic and control groups. In the colon, ITAI significantly decreased IL-8 and IL-10 concentrations (*P* < 0.05), but had little effect on IFN-γ and TNF-α concentrations (Fig. 3b). For Ig (sIgA and IgG), in the jejunum, ITAI significantly decreased (*P* < 0.05) sIgA and IgG concentrations (Fig. 3a). In the colon, the concentration of sIgA from the antibiotic group was significantly decreased (P < 0.05) compared to control group (Fig. 3b). These results suggest that ITAI affected jejunal and colonic mucosal immune responses with a decrease in specific cytokines and Igs concentrations.

### Immunoglobulin and cytokines concentrations in plasma

**Fig. 4:**
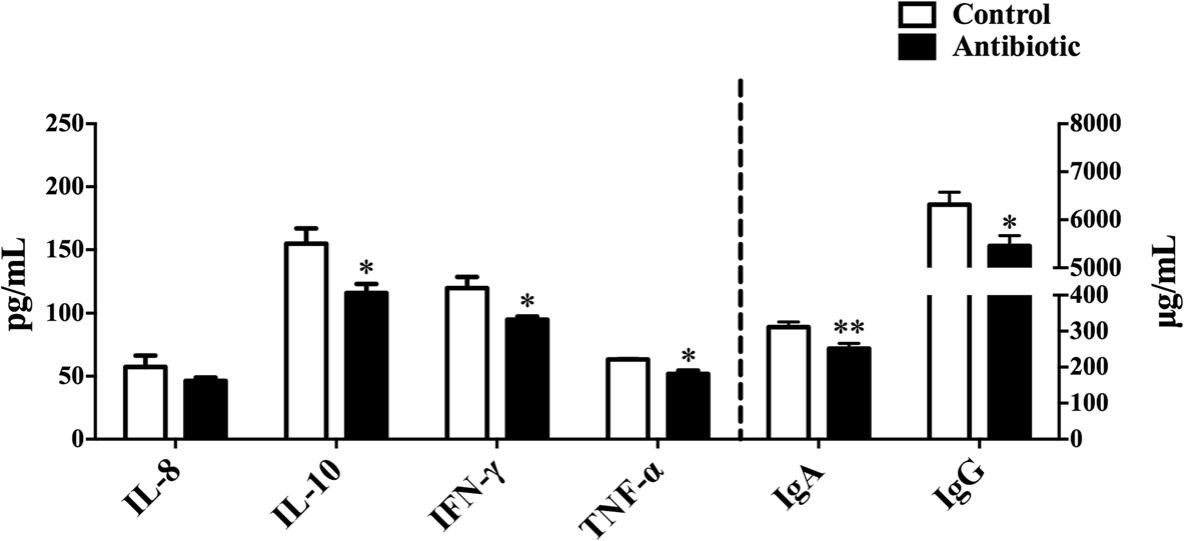
Effect of ileum terminal antibiotic infusion (ITAI) on the concentrations of serum cytokines and immunoglobulins in plasma of growing pigs. Values are means ±SEMs (*n* = 6). Asterisks indicate statistically significant difference from control: *P < 0.05, **P < 0.01. IL-8 = interleukin-8; IL-10 = interleukin-10; IFN-γ = interferon-γ; TNF-α = tumor necrosis factor-α; IgA = immunoglobulin A; IgG = immunoglobulin G

To understand the effects of ITAI on the systemic immune response, serum cytokines and Igs concentrations were determined by ELISA (Fig. 4). For cytokines, ITAI significantly decreased (*P* < 0.05) IL-10, IFN-γ and TNF-α concentrations, while IL-8 concentration remained similar in both experimental groups. Pigs in the antibiotic group recorded lower (*P* < 0.05) IgG and IgA concentrations. These results suggest that ITAI affected serum immune responses with a decrease in serum specific cytokines and Igs concentrations in growing pigs.

### Correlation analysis of immune markers levels in the jejunal and colonic mucosa and serum, dominant microbial communities counts in the jejunum and colon and SCFA concentration in the colon

**Fig. 5:**
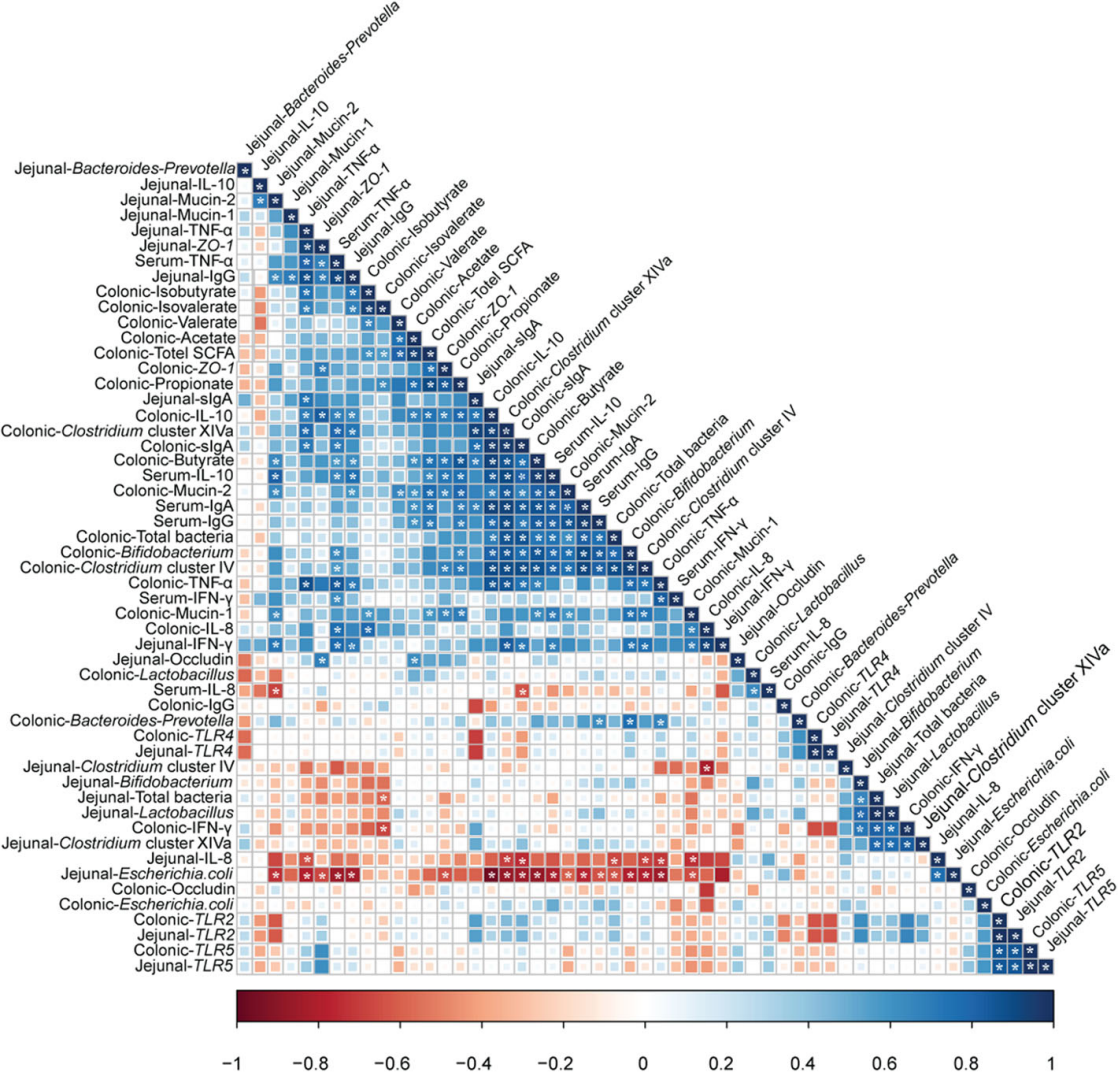
Correlation analysis of immune markers levels in the jejunal and colonic mucosa and serum, dominant microbial communities counts in the jejunum and colon and SCFA concentration in the colon. The *R* package of “corroplot” was used for generating the heat maps. The blue represents a significant positive correlation, and the red represents a significant negative correlation. Asterisks indicate statistically significant difference from control: *P < 0.05. Total SCFA = total short-chain fatty acid; *TLR2* = toll-like receptor 2; *TLR4* = toll-like receptor 4; *TLR5* = toll-like receptor 5; *IL-8* = interleukin-8; *IL-10* = interleukin-10; *IFN-γ* = interferon-γ; *TNF-α* = tumor necrosis factor-α; *ZO-1* = zonula occludens-1; sIgA = secretory immunoglobulin A; IgG = immunoglobulin G

The correlation analysis of all measured bacterial taxa counts, immune parameters levels and SCFA concentration has been shown in Fig. 5. The scatterplots of some correlations were shown in the Additional file 1: Figure S1. The correlation analysis revealed that *Clostridium* cluster IV and *Clostridium* cluster XIVa counts were positively correlated (*P* < 0.05) with butyrate concentration in the colon. *Clostridium* cluster IV and *Clostridium* cluster XIVa counts in the colon were positively correlated (*P* < 0.05) with colonic IL-10 and sIgA concentrations, serum IL-10, IgA and IgG and jejunal sIgA. *Clostridium* cluster XIVa counts in the colon were positively correlated (*P* < 0.05) with jejunal TNF-α and IgG concentrations. *Bifidobacterium* counts in the colon were positively correlated (*P* < 0.05) with colonic IL-10 and sIgA concentrations and serum IL-10, IgA and IgG. Butyrate concentration was positively correlated (*P* < 0.05) with mRNA expression levels of Mucin-1 and Mucin-2 in the colon. Colonic sIgA and IL-10 concentrations showed positive correlations (*P* < 0.05) with serum IgA, IgG, IL-10 and TNF-α concentrations, and jejunal sIgA concentration. In the jejunum, the counts of *Escherichia coli* were negatively correlated (*P* < 0.05) with the concentrations of IgG and TNF-α. These results suggest that the changes in intestinal microbiota are correlated with alterations of Ig and cytokines concentrations. Furthermore, colonic Ig and cytokines were correlated with serum and jejunal Ig and cytokines.

## Discussion

Previous studies have shown that oral antibiotics may cause gut microbiota dysbiosis, suppress the innate immune defenses, and lead to the increased pathogen colonization and disease susceptibility in mice [18, 28, 29]. For the first time, to our knowledge, the current experiment evaluating the effect of ITAI on specific bacterial composition and mucosal immune response in the jejunum and colon, and serum immune index of pigs. Our findings suggest that ITAI affected specific bacteria in the colon, and altered some markers of immunity in the colon, serum and jejunum, which may increase opportunities for *Escherichia coli* to colonize in the jejunum.

The present study showed that ITAI significantly changed the specific intestinal bacteria, with a decrease in generally beneficial bacteria (*Bifidobacterium*, *Clostridium* cluster IV and *Clostridium* cluster XIVa) in the colon and an increase in *Escherichia coli* in the jejunum (Fig. 1). *Bifidobacterium* is reported to be a health-promoting bacterium and potential probiotic [30]. One of the most important functions of *Bifidobacterium* is to reduce TLR4 signalling by competing for nutrients and adhesion site with gram-negative pathogens, and then promoting the intestinal mucosal barrier function and regulating intestinal immunity [31, 32]. Butyrate-producing bacteria, such as *Clostridium* cluster IV and *Clostridium* cluster XIVa, together with butyrate, could benefit colonic homeostasis by promoting epithelial energy metabolism and modulating immune development [33]. In the present study, another finding was that ITAI increased *Escherichia coli* counts in the jejunum (Fig. 1). The increase in *Escherichia coli* has been shown to be associated with the development of Crohn’s disease [34]. However, the exact cause of higher *Escherichia coli* counts in the antibiotic group compared with control group is not clear. Many studies have shown that exposure to antibiotics can cause gut microbiota dysbiosis and inflammatory bowel disease [35, 36]. The loss of bacterial ligands, alterations in the metabolites and the loss of specific bacterial signals with antibiotic treatment may induce changes of immune function, leading to increased pathogen colonization and disease susceptibility [37]. A previous study showed that treatment with oral metronidazole affects the expression of Mucin-2, reduces the integrity of the mucous layer and increases mucosal attachment of *Citrobacter rodentium* in the colon of rats [1]. Therefore, in the present study, ITAI may affect gut immunity, leading to a vulnerable gut environment which could be susceptible to pathogen colonization and disease. A significant negative correlation between the concentrations of IgG and TNF-α and the counts of *Escherichia coli* (Fig. 5) suggests that there may be the potential causal relation between the increase in *Escherichia coli* and the change in immune system in the jejunal mucosa.

The three major SCFAs acetate, propionate and butyrate are main end products from carbohydrates fermentation, and are less produced from amino acids in hindgut, while branched chain fatty acids (BCFAs) (i.e., isobutyrate and isovalerate) originate exclusively from the breakdown of protein [38, 39], and represent good markers of protein breakdown in hindgut. In the present study, ITAI decreased SCFA concentration in the colon (Table 4), especially propionate and butyrate. SCFA create a slightly acidic environment, thereby preventing the growth of acid-sensitive detrimental bacteria, including pathogenic *Salmonella* and *Escherichia coli* [40]. Butyrate is a major energy source for colonocytes [41]. Furthermore, propionate and butyrate exert an anti-inflammatory function [42, 43], which are vital in maintaining gut tolerance. The low propionate and butyrate concentrations in the antibiotic group may not benefit gut health. We also found antibiotic infusion decreased the concentrations of isobutyrate and isovalerate concentrations (Table 4). It suggests that ITAI may decrease incomplete degradation of amino acids (especially leucine and valine) or BCFA-producing bacteria counts in the colon. A previous study indicates that BCFAs may be able to promote intestinal integrity [44].

Intestinal microbiota and its metabolites could affect systemic and intestinal immunity. In the present study, ITAI decreased intestinal pro-inflammatory cytokines levels, such as IL-8 in the colonic mucosa, IFN-γ and TNF-α in the serum and jejunal mucosa (Figs. 2, 3 and 4). Previous studies have revealed pro-inflammatory cytokines, whose expression was affected by intestinal commensal bacteria, play a central role in intestinal inflammatory disease [45, 46]. Anti-inflammatory cytokines, such as IL-10 and IL-4, prevent over-activation of immune response and suppress the production of pro-inflammatory cytokines to maintain immune homeostasis [47]. At present, we observe that ITAI decreased IL-10 level in the colon (Figs. 2 and 3). A previous study reported that colonization with a mixture of *Clostridium* species from clusters IV and XIVa can suppress colitis through the induction of IL-10-producing regulatory T (Treg) cells [48]. A significant positive correlation between IL-10 expression and the counts of *Clostridium* clusters IV and XIVa in the colon (Fig. 5) indicates that the reduction in the counts of *Clostridium* clusters IV and XIVa may partly contribute to the changes in the colonic IL-10 level in the antibiotic group. Cytokines play a crucial role in the immune and inflammatory responses, and their balance is important for protection against infection [49]. Previous studies showed that several cytokine knockout models in mice failed to resist pathogen infection [50, 51]. In the present study, the reduced pro-inflammatory and anti-inflammatory cytokine with antibiotic treatment may increase susceptibility to infection by pathogens.

The intestinal barrier is mainly formed by a layer of epithelial cells joined together by tight junctions. It acts as a critical line of defense against pathogenic agents and luminal antigens [52]. In the present study, downregulation of intestinal barrier genes expression in the jejunal and colonic mucosa, such as *MUC1*, *MUC2* and *ZO-1* (Fig. 2) suggest that ITAI may decrease the integrity of the mucous layer, consequently generating a vulnerable gut environment which could be susceptible to pathogen infection. However, the underlying mechanism of intestinal barrier genes downregulation is unknown. A previous study has shown that antibiotic treatment led to the loss of normal microbiota subsets, thus reducing epithelial exposure to microbiota-derived microorganism-associated molecular patterns (MAMPs). Fewer MAMPs with antibiotic intervention resulted in the decreased pattern recognition receptors stimulation [37], which may lead to the decreased intestinal barrier function and integrity. Furthermore, butyrate, the most extensively studied SCFA, has been shown to maintain the gut integrity [53]. A correlation analysis revealed that butyrate concentration was positively correlated with mRNA expression levels of *MUC1* and *MUC2*. Therefore, the low butyrate concentration in the antibiotic group may also lead to downregulation of intestinal barrier genes expression. However, histology and immunohistochemistry are still needed to validate down-regulation of intestinal barrier genes further.

ITAI also decreased systemic and intestinal Ig concentrations. The present study identified a decrease in the concentrations of sIgA and IgG in the jejunal mucosa and sIgA in the colonic mocosa in the antibiotics group (Fig. 3). Secretory immunoglobulin A, secreted by plasma cells existing in intestinal lamina propria, forms a major component of the local immune barrier of the intestine and plays an integral role in intestinal protection [54]. Therefore, the level of sIgA in the intestine was used as an indicator to evaluate intestinal mucosal immunity. In addition, IgG also participates in the process of intestinal immune response [55, 56]. The low concentrations of intestinal Igs in the antibiotic group may indicate a decrease in defensive capacity of the mucosal immune system. ITAI also decreased IgG and IgA concentrations in the serum (Fig. 4). Actually, IgG and IgA are key components of the humoral immunity in all mammals, which are the major serum immunoglobulins that protect the extravascular compartment against microorganisms [57]. Lower IgG and IgA concentrations in serum of the antibiotic group may indicate a decrease in humoral immunity. A previous study reported that *Bifidobacterium* can induce systemic and intestinal IgA production [58, 59]. Furthermore, as shown by gnotobiotic studies, the production of IgA is stimulated by the colonization of clostridia [60].Correlation analysis revealed a correlation between colonic sIgA and serum IgA level and the counts of *Bifidobacterium* and *Clostridium* clusters IV and XIVa in the colon (Fig. 5), suggesting that the decrease in colonic sIgA and serum IgA level in the antibiotic group may partly be due to the change in the counts of *Bifidobacterium* and *Clostridium* clusters IV and XIVa. ITAI caused similar innate immune-related responses as those observed in other models of antibiotic-induced dysbiosis [61]. These changes may represent a general adaptive pattern of the host in response to alterations in the composition of the microbiota.

However, why ITAI affected immune system in the jejunal mucosa is not clear. Correlation analysis revealed that colonic immunoglobulins and cytokines were correlated with serum and jejunal Ig and cytokines. These results indicate that the change in immunity in the colon may partly result in alteration of immune system in the jejunum. As mentioned earlier, in rat models of acute and chronic colitis, increased levels of pro-inflammatory cytokines were demonstrated in distant and intact segments of the small intestine without any significant histological changes [62].

In the present study, IATI directly disrupted the hindgut bacteria, reducing SCFA-produing bacteria counts and SCFA concentration in the colon. Our previous studies showed that oral antibiotics had limited effects on microbiota and SCFA concentration in the large intestine [13, 20]. This may be due to the gradual dilution of antibiotics in the gut, which led to the weakened effects. Oral antibiotics or ITAI may induce the loss of bacterial ligands, alterations in the metabolites and the loss of specific bacterial signals, altering immune response with a decrease in the levels of specific inflammatory cytokines, gene expression and immunoglobulins in the intestinal mucosa and serum.

## Conclusions

ITAI affected specific jejunal and colonic microbial population and immune status, and serum immune index. ITAI increased *Escherichia coli* counts in the jejunum, while reducing generally beneficial bacteria counts in the colon; Moreover, ITAI affected intestinal and systemic immunity, as evidenced by a decrease in the levels of specific inflammatory cytokines and immunoglobulins in the intestinal mucosa and serum. Correlation analysis revealed that the change in intestinal microbiota was correlated with alterations of Igs and cytokines concentrations. Compared with oral antibiotics (primarily disrupt foregut microbiota), the findings aided in understanding of the impact of ITAI (disrupts hindgut microbiota) on gut and systemic immune in growing pigs.

## Additional files

Additional file 1: **Figure S1.** Scatterplots demonstrating correlations of some immune markers levels in the jejunal and colonic mucosa and serum, dominant microbial communities counts in the jejunum and colon and butyrate concentration in the colon. *IL-8* = interleukin-8; *IL-10* = interleukin-10; *IFN-γ* = interferon-γ; *TNF-α* = tumor necrosis factor-α; sIgA = secretory immunoglobulin A; IgG = immunoglobulin G. (DOCX 1289 kb)

## Abbreviations

IFN-γ: Interferon-γ
IL: Interleukin
ITAI: Ileum terminal antibiotic infusion
MUC1: Mucin-1
MUC2: Mucin-2
OCLN: Occludin
SCFA: Short-chain fatty acid
sIgA: Secretory immunoglobulin A
TLR: Toll-like receptor
TNF-α: Tumor necrosis factor-α
ZO-1: Zonulaoccludens-1
