# Supplementary material for: Ileum terminal antibiotic infusion affects jejunal and colonic specific microbial population and immune status in growing pigs
Source: J Anim Sci Biotechnol. 2018 Jul 2;9:51. doi: 10.1186/s40104-018-0265-x (PMC6027559; doi:10.1186/s40104-018-0265-x)
Supplement: Supplementary file 1 — Figure S1. Scatterplots demonstrating correlations of some immune markers levels in the jejunal and colonic mucosa and serum, dominant microbial communities counts in the jejunum and colon and butyrate concentration in the colon. IL-8 = interleukin-8; IL-10 = interleukin-10; IFN-γ = interferon-γ; TNF-α = tumor necrosis factor-α; sIgA = secretory immunoglobulin A; IgG = immunoglobulin G. (DOCX 1289 kb) [file 40104_2018_265_MOESM1_ESM.docx]

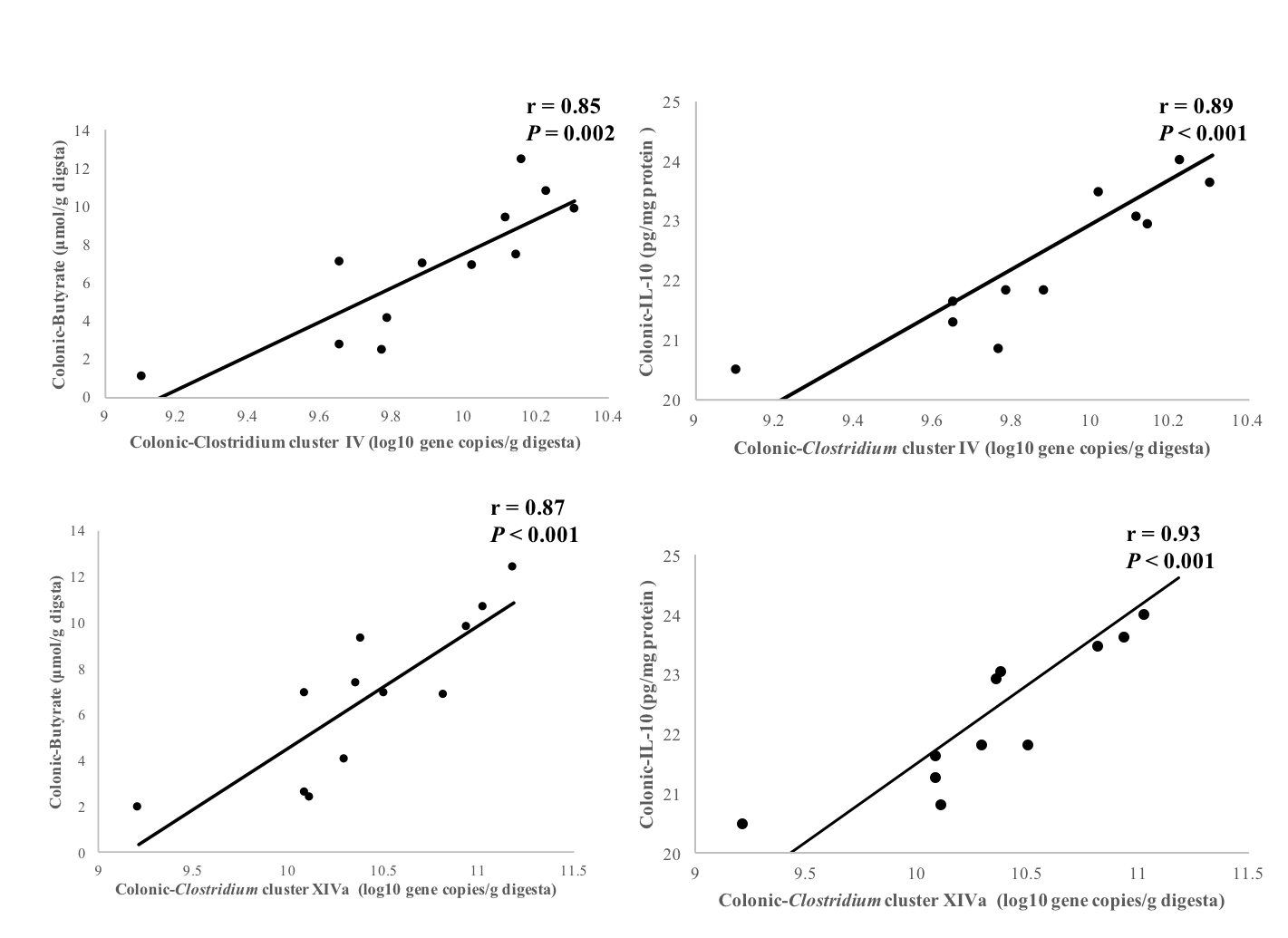

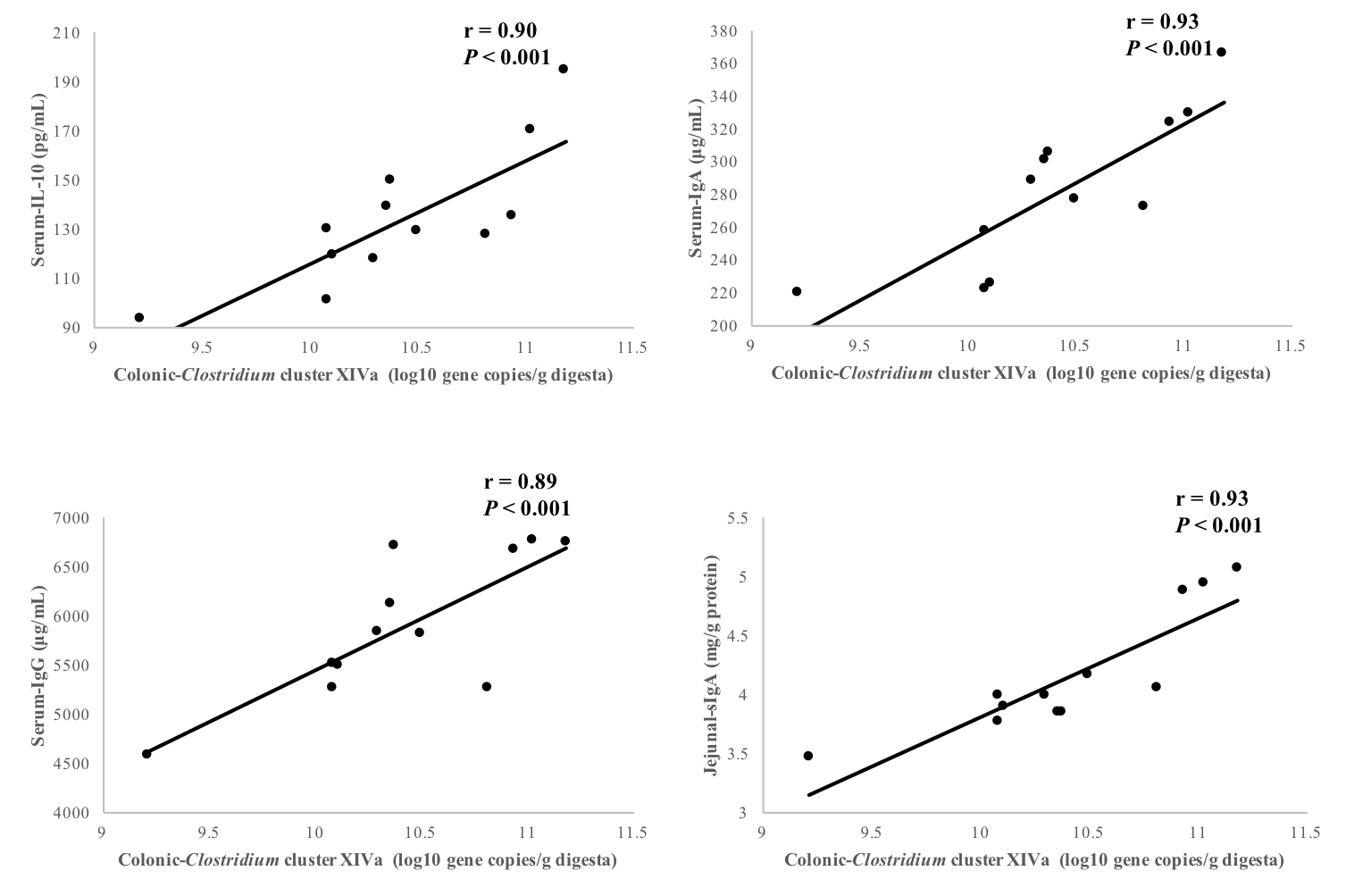


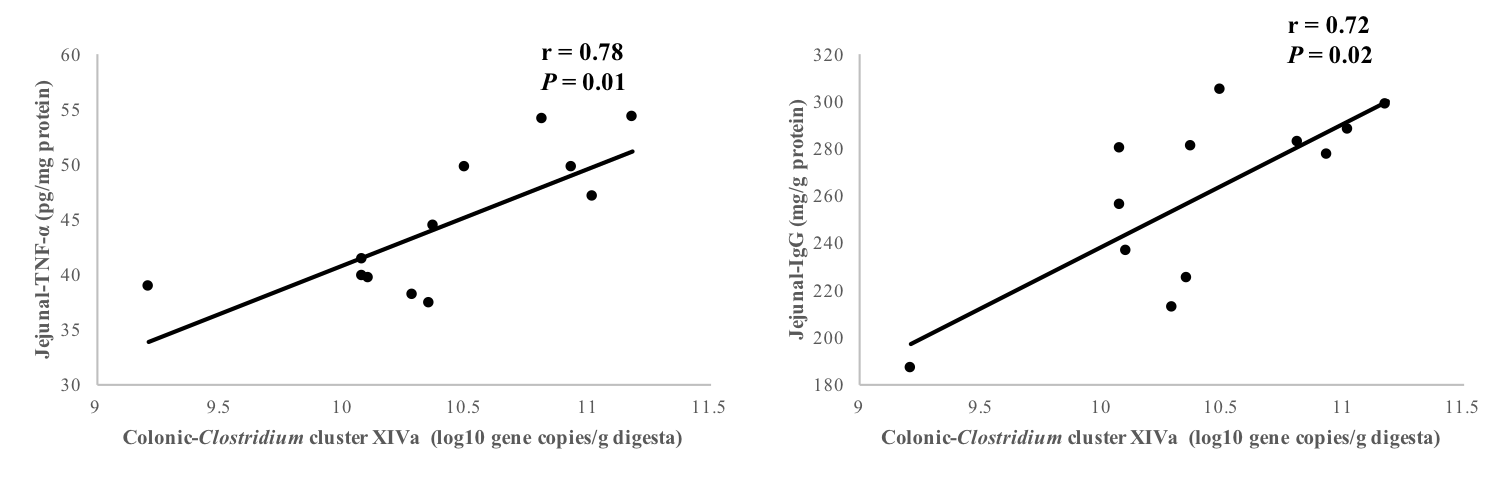

**Fig.S1** Scatterplots demonstrating correlations of some immune markers levels in the jejunal and colonic mucosa and serum, dominant microbial communities counts in the jejunum and colon and butyrate concentration in the colon. *IL-8* = interleukin-8; *IL-10* = interleukin-10; *IFN-γ* = interferon-γ; *TNF-α* = tumor necrosis factor-α; sIgA = secretory immunoglobulin A; IgG = immunoglobulin G.
